# CD20 Antibody Primes B Lymphocytes for Type I Interferon Production

**DOI:** 10.1371/journal.pone.0067900

**Published:** 2013-06-18

**Authors:** Dongsheng Xu, Andrew Staedman, Luwen Zhang

**Affiliations:** 1 School of Biological Sciences, University of Nebraska, Lincoln, Nebraska, United States of America; 2 Nebraska Center for Virology, University of Nebraska, Lincoln, Nebraska, United States of America; University of Illinois at Chicago, United States of America

## Abstract

CD20 is a B cell surface marker that is expressed in various stages in B lymphocytes and certain lymphomas. Clinical administration of CD20 antibody, such as rituximab, is used widely to treat human B-cell lymphomas and other diseases. However, CD20 antibody failed to treat systemic lupus erythematosus (SLE or lupus). The reason for the failure is currently unknown. Type I interferons (IFN) are a major component for the host innate immunity, and a key pathogenic factor in lupus. We found that CD20 antibody potentiated human B cells for its production of IFNs *in vitro*. This function was specific to CD20-expressing cells and the potentiation function seems to be instant. In addition, ectopic expression of CD20 in non-B-lymphocytes increased the IFN promoter reporter activities. Because IFNs are a key pathogenic factor in lupus, our data suggest that, in the presence of virus infection, the CD20-antibody-mediated enhancement of IFN production might be related to its failure in lupus treatments. This work may provide new insights for CD20-Ab therapeutic applications.

## Introduction

Systemic lupus erythematosus (SLE), also called lupus, is a chronic systemic autoimmune disease that affects about 0.1% of the US population, and results in inflammation and damage to a range of organ systems including joints, muscles and other parts of the body.

Human type I Interferons (IFN) consist of 13 distinct IFN-α and other subtypes [1,2]. IFNs are apparently a hallmark in lupus. IFN levels and IFN-stimulated genes, collectively called IFN signatures in some of the literature, are elevated in lupus patients [3–8]. The use of IFNs for the treatment of other diseases has caused lupus-like syndromes [9,10]. In rodent models of lupus, mice have failed to develop lupus manifestations if the IFN receptor is deleted [11]. IFN promotes survival and differentiation of mature lymphocytes, class switching at immunoglobulin heavy chain loci, and activation of dendritic cells (DC) [12]. Finally, IFN enhances the activation of B lymphocytes by RNA-associated autoantigens [13]. Thus, the IFN pathway has emerged as a focal point for understanding mechanisms of autoimmunity in lupus.

CD20 is a 33–37 kDa membrane-associated and non-glycosylated phosphoprotein expressed on the surface of all mature B-cells [14,15]. CD20 plays a role in the development and differentiation of B-cells into plasma cells. The CD20 protein has no known natural ligand and its function is very elusive [14,15]. It is suspected that CD20 acts as a calcium channel in the cell membrane [16]. In addition, recent data suggest that CD20 may play a central role in the generation of T cell-independent antibody responses [17].

The CD20 antibodies, such as rituximab, Ibritumomab tiuxetan, and tositumomab, are all active agents in the treatment of some B cell lymphomas and leukemias [18,19]. Interestingly, recent randomized placebo-controlled trials failed to demonstrate the efficacy of Rituximab in patients with SLE [20–23]. Many reasons might explain the failure, such as the small number of patients, the relatively short follow-up time, and the use of relatively high doses of other medicines [24]. Others suggested that anti-inflammatory strategies, not just B cell depletion, may be required for optimal therapy for SLE [25].

We were testing if the CD20-Ab affects Epstein–Barr virus (EBV)-mediated transformation of human B lymphocytes, and in the process, we found that CD20-Ab, or rituximab, potentiated B lymphocytes for the production of IFNs. This work suggested that CD20 might be a component of innate immunity in B lymphocytes. Because IFN is a key pathogenic determinant for lupus [3,26–28], the potentiation of B lymphocytes for IFN production might be related to the failure of the lupus treatment with the antibody [20–23].

## Materials and Methods

### *Plasmids, viruses, and antibodies*

CD20 expression plasmid was purchased from Addgene (Plasmid 1890). The IFN-β-promoter reporter constructs were gift from Dr. Rutuan Lin. Sendai virus stock was purchased from Spafas, Inc. For virus infection, 200 HA units/ml Sendai virus were added to the target cells for 6 h, and cells were then collected for RNA isolation. Vesicular stomatitis virus (VSV), Indiana strain, was a gift from Dr. Asit Pattnaik. Rituximab (CD20 antibody) was purchased from Genetech. Anti-Sendai virus antibody was purchased from U.S. Biological (Cat#: S0700).

### *Cell Culture, Transient Transfection, and Reporter Assays*

293T is a human fibroblast line, and was grown in Dulbecco’s modified Eagle medium (DMEM, Gibco BRL) supplemented with 10% fetal bovine serum (FBS; Gibco BRL) and 1% Penicillin-streptomycin (PS) at 37 °C in 5% CO_2_ incubation. DG75, IB4 and LCL are all B cell lines. THP1 is a monocyte line and Jurkat is a T cell line. All those cells were maintained in RPMI-1640 plus 10% FBS. Effectene (Qiagen) was used for the transfection of 293T following Manufacturer’s recommendation. The luciferase reporter assays were performed using the assay kit from Promega according to manufacturer’s recommendation.

### *RNA Extraction and RNase Protection Assays *(*RPA*)

Total RNA was isolated from cells using the RNeasy total RNA isolation kit (Qiagen, Valencia, CA) or TRIzol extraction methods. RPA was performed with 10 µg of total RNA using the RNase protection assay kit II (Ambion, Houston, TX) at 55 °C [29–31]. Sometimes, gradient temperatures were performed for RPA when difficulties in RPA were encountered [32]. The GAPDH probe was purchased from U.S. Biochemicals. The probe for IFN-β was a gift from Dr. Ganes Sen.

### *Western Blot Analysis with Enhanced Chemiluminescence *(*ECL*)

Separation of proteins on SDS-PAGE was carried out following standard protocol. After the proteins were transferred to a nitrocellulose or Immobilon membrane, the membrane was blocked with 5% nonfat dry milk in TBST (50 mm Tris-HCl, pH 7.5, 200 mm NaCl, 0.05% Tween 20) at room temperature for 10 min. It was then washed briefly with TBST and incubated with the primary antibody in 5% milk in TBST for 1 h at room temperature or overnight at 4 °C. After washing with TBST three times (10 min each), the membrane was incubated with the secondary antibody at room temperature for 1 h. It was then washed three times with TBST, treated with ECL detection reagents (Amersham Biosciences), and exposed to Kodak XAR-5 film.

### *IFN-α Measurement*

The concentration of IFN-α was determined by a commercially available human interferon α (Hu-IFN-α) ELISA kit (PBL Biomedical Laboratories; catalog number 41100) according to the manufacturer’s recommendations. The kit is able to detect human IFN-αA, IFN-α2, IFN-αA/D, IFN-αD, IFN-αK, and IFN-α4b. However, it cannot detect IFN-β, IFN-ω, and other IFN-α subtypes. Samples were examined in duplicates.

## Results

### *Rituximab potentiates B lymphocytes for IFN productions*

**Figure 1:**
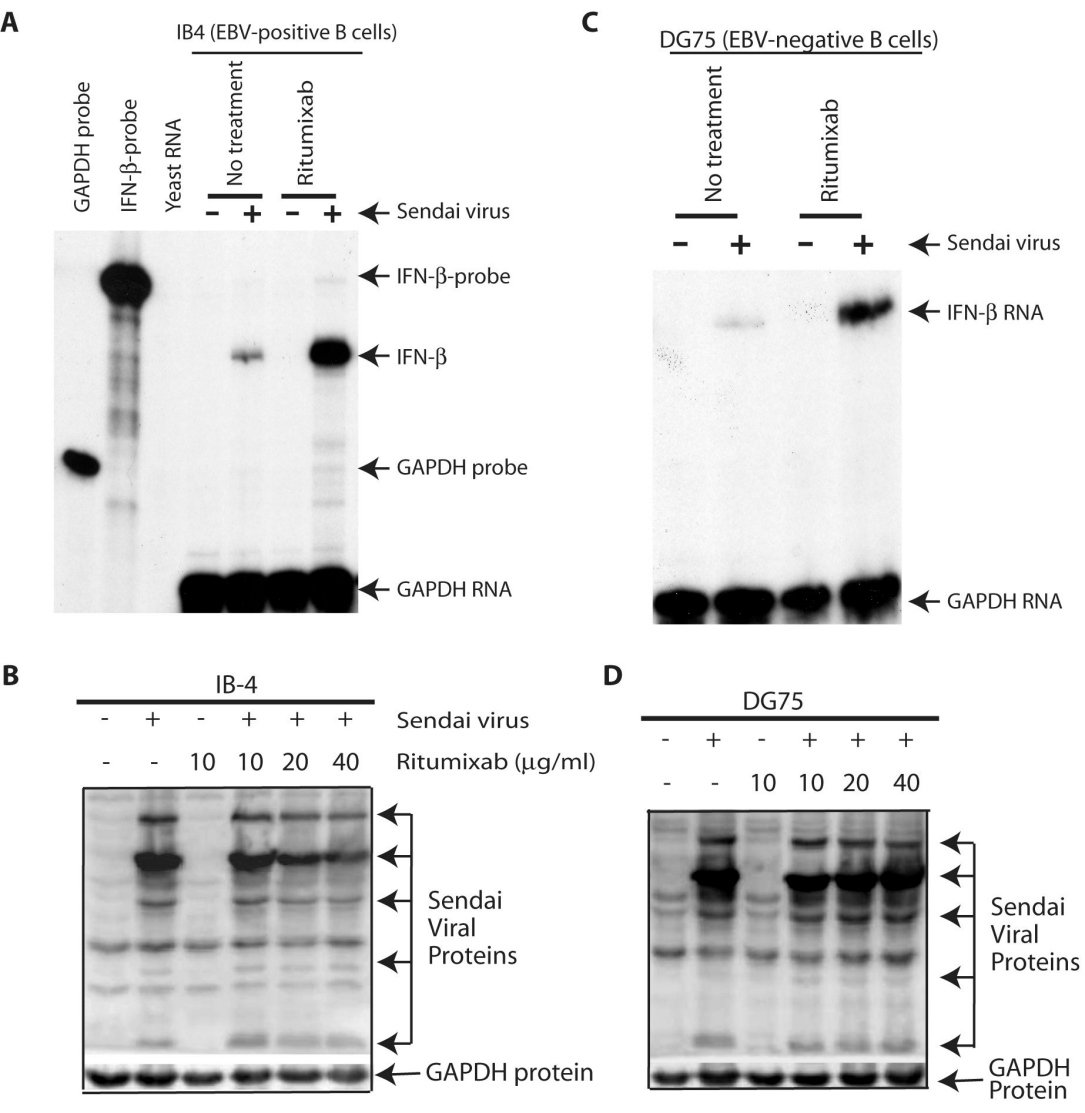
Rituximab enhances cells for the production of IFN-β. A. IB-4 is a commonly used EBV transformed B cells. The cells were treated with rituximab (10 mg/ml) and at the same time, were infected by Sendai virus (200 HA units/ml) for 6 h. Total RNAs were isolated and used for RPA with IFN-β and GAPDH probes. Yeast RNA was used as negative control. Specific protections of IFN-β and GAPDH RNAs are indicated. B. Sendai virus protein expression. Different amounts of Rituximab and constant. Sendai (200 HA units/ml) were used to treat cells simultaneously for 6 hours. Cell lysates were used for detection of viral replication. Specific viral proteins and GAPDH are as shown. C and D: DG75 cells are EBV-negative Burkitts’ lymphoma cells and were treated with Rituximab and Sendai virus simultaneously for 6 hours. IFN-β productions were measured in C, and Sendai viral protein expression was determined in D.

Rituximab is a humanized antibody against CD20, and it is used successfully for the treatment of B lymphomas. We suspect that the CD20 antibody may affect B cell biology and therefore affect the production of IFNs upon viral infection. IB4 is a commonly used B cell line transformed by EBV *in vitro* [33–38]. Rituximab (10µg/ml) was used to treat cells and at the same time, the Sendai virus was used to infect the cells. The use of 10µg/ml Rituximab is common in the field [39–41]. As shown in the Figure 1A, IFN production was enhanced when the CD20-Ab was used. Type I IFNs have multiple subtypes [42]; however, the use of IFN-β as an indicator for type I IFNs production is well-established and appreciated in the field. To eliminate the possibility that Rituximab enhances the viral replication and thus the IFN production, we did examine the viral replication by detection of the Sendai viral protein expression. As shown in Figure 1B, the expression of viral protein was not enhanced with the treatment of CD20 antibody (Figure 1B). To eliminate the effect of EBV in the enhanced IFN production, we have used other B cell line that lack of EBV infection. DG75 is an EBV-negative Burkitts’ lymphoma line. As shown in Figure 1C, IFN production was also enhanced, and the viral protein expressions were not increased upon the CD20 Ab treatments (Figure 1D). Therefore, CD20-Ab enhances cells for the production of IFNs upon viral infection.

### *The effects of Rituximab on IFN productions is specific for B lymphocytes*

**Figure 2:**
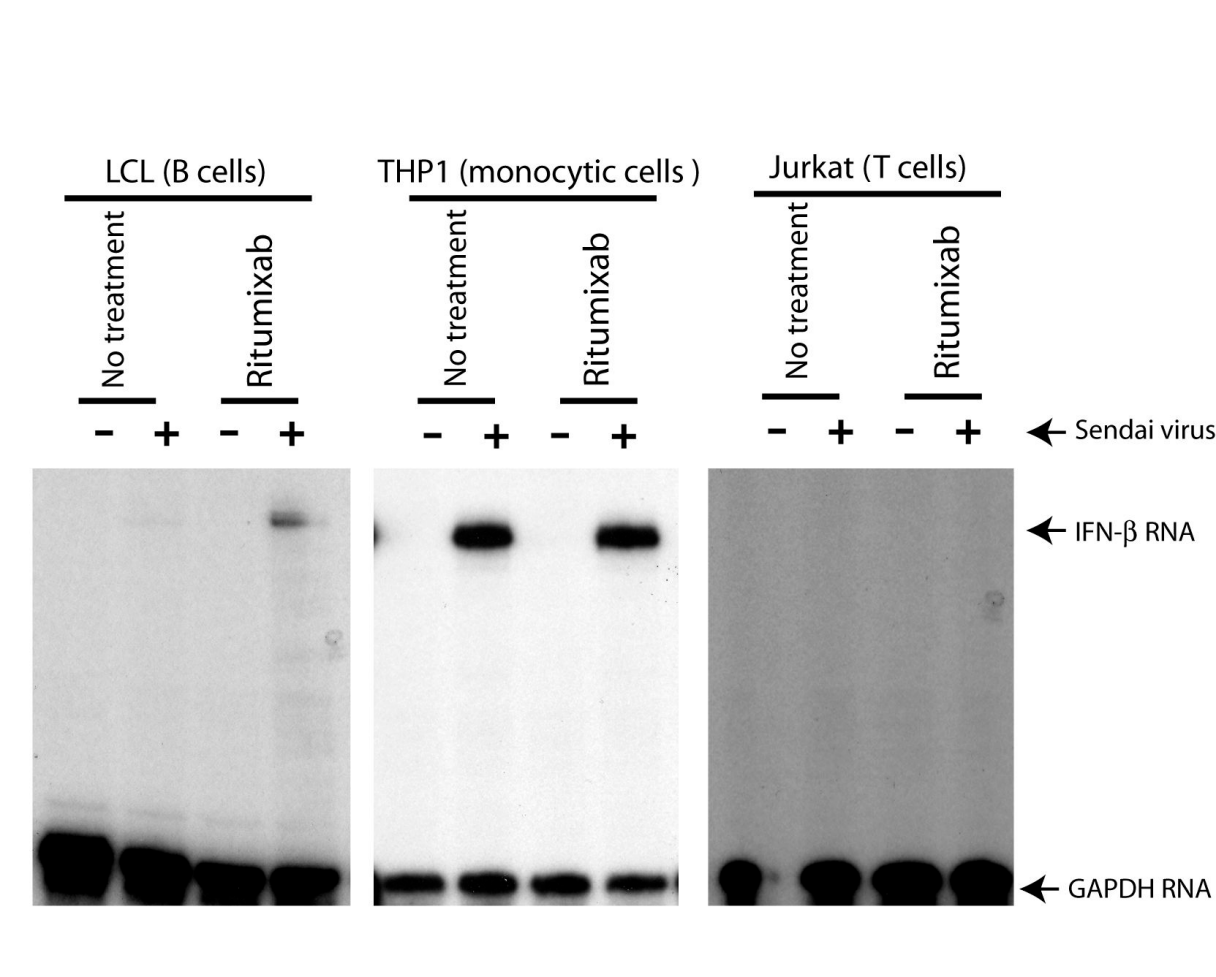
The specificity of the Rituximab treatment for IFN enhancement. LCL is another EBV-transformed B cell line. THP-1 is a human acute monocytic leukemia cell line; and Jurkat is an immortalized line of T lymphocyte cells. All these cells were treated with Rituximab and Sendai virus simultaneously for 6 hours. IFN-β productions were measured. Specific protections of IFN-β and GAPDH RNAs are indicated.

To test if the potentiation effect of the CD20 Ab is B lymphocyte specific, we have treated several cell lines with different cell lineages. LCL is another EBV-transformed B lymphocytes at early passages, and no mutation are expected for the line. Jurkat is a T cell line and THP1 is a monocyte line. The same experimental procedures were used for those lines, and Sendai virus was used for induction of IFNs. As shown in Figure 2, while CD20 has potentiation effects on LCL, the effects were not present for THP1 and Jurkat cells. Therefore, the effects of Rituximab on IFN productions are likely specific for B lymphocytes.

### *Time and dosage effects on Rituximab mediated effects on IFN productions*

**Figure 3:**
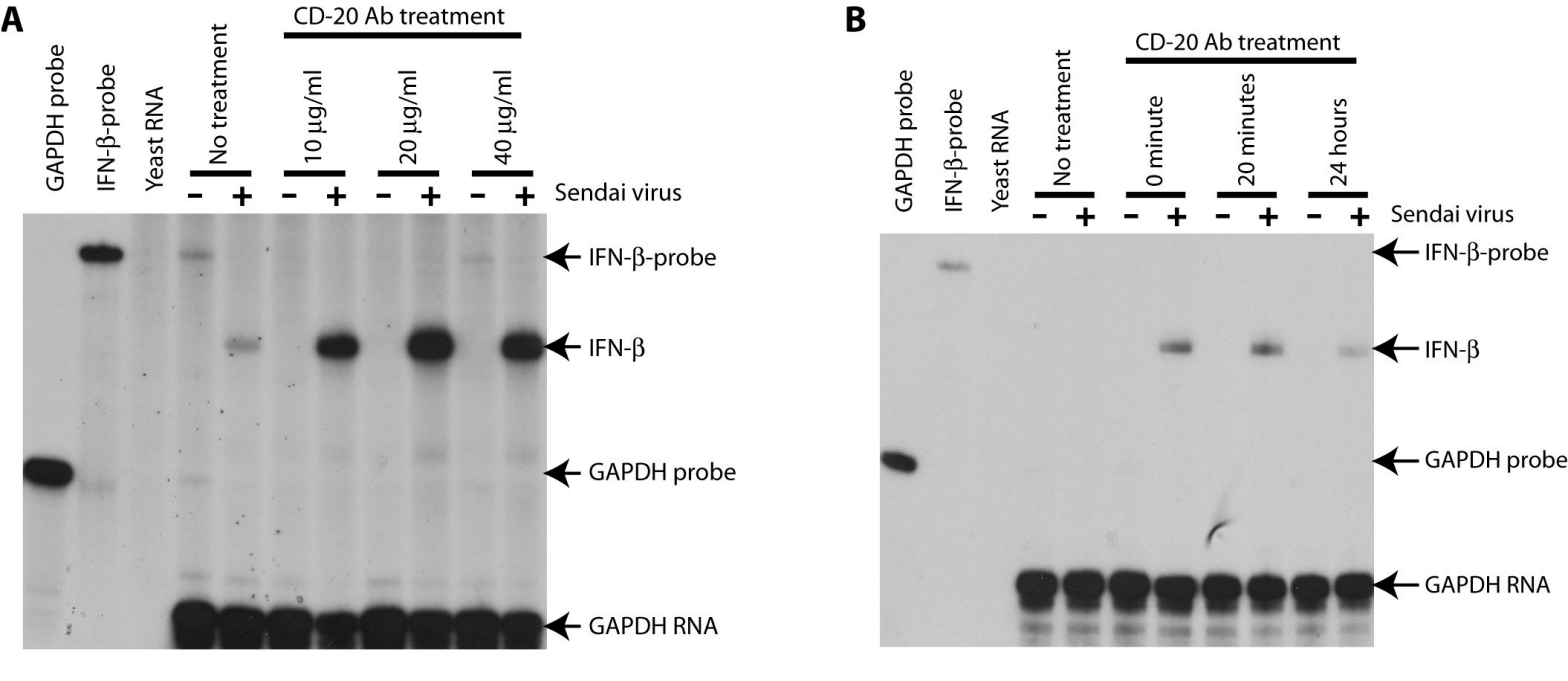
Dose and time-dependent enhancement of the IFN-production by Rituximab. A. Different amounts of Rituximab and constant Sendai virus (200 HA units/ml) were used to treat IB-4 cells simultaneously. The RNA was isolated after 6 hours and RPA was used for detection of IFN productions. B. the IB4 cells were treated with 10 mg/ml Rituximab with indicated time, Sendai was then used to infect the cells. The RNA was isolated after 6 hours and RPA was used for detection of IFN productions. Specific protections of IFN-β and GAPDH RNAs are indicated.

We further examined dose and time requirements for the enhancement. Different amounts of CD20 Ab were used with Sendai virus simultaneously. As shown in Figure 3A, there seemed to be a dose response to the CD20-Ab. However, the dosage of 10µg/ml, commonly used in the field [39–41], is sufficient to enhance IFN production. In addition, the CD20-Ab was used to treat cells for various times, then infect with Sendai virus and the RNA were isolate 6 hours later for IFNs detection. As shown in Figure 3B, longer time exposure to the CD20-Ab is actually detrimental for the enhancement. The reduction might be related to apoptosis as the CD20-Ab treatment may induce apoptosis [43]. The data also suggest that the enhancement of IFN production is likely to be an early event in Rituximab treatment.

### *CD20 expression enhanced IFN activation*

**Figure 4:**
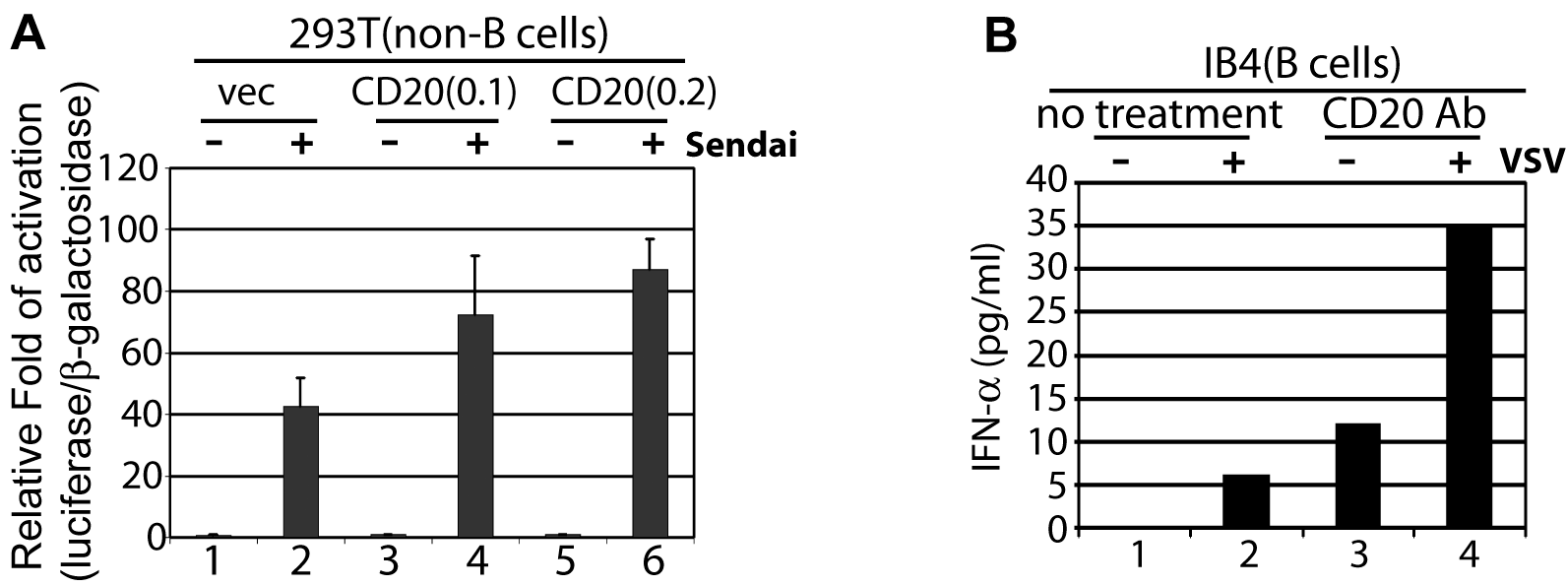
A. CD20 expression enhances IFN promoter reporter activity. 293T cells were transfected with IFN-β promoter reporter constructs and expression plasmid of CD20 (0.1, and 0.2 mg). After the overnight incubation, Sendai virus (20 HA units/ml) was added and the luciferase and β-galactosidase activities were measured 24 hours later. The relative fold of activation was calculated as a ratio of luciferase over β-galactosidase activities. Standard deviations are as shown. **B**. **Rituximab enhances VSV-induced IFN-α production**. IB4 cells were treated with VSV (M.O.I=1) and Rituximab. 24 hours later, medium was collected for ELISA for IFN-α (multiple subtypes) measurements. Samples were measured in duplications, and the average of duplications is as shown.

To test if CD20 expression in non-B lymphocytes would enhance these cells for IFN production, we have transfected CD20 expression plasmid into 293T cells along with IFN-β-promoter reporter construct. As shown in Figure 4A, while CD20 itself has limited effect on the promoter activity, the Sendai virus induced activation of the promoter reporter was enhanced, in agreement with the previous data.

### *Other virus can also induce IFN production*

The Sendai virus was chosen because it is the most commonly used virus for IFN induction studies. The use of Sendai virus for IFN production was well established and appreciated in the field. However, to avoid the possibility that the potentiation effect of CD20Ab was a virus specific phenomenon, we tested whether the CD20-Ab enhanced another virus-induced IFN production. Vesicular stomatitis virus (VSV) was used and it is known the virus activates IFN pathway through toll-like receptor 7 (TLR7) pathway [44]. VSV infect B cells poorly, so we used ELISA to monitor the IFN production after 24 hours of infection. As shown in Figure 4B, CD20-Ab enhances the production of IFNs by VSV. Surprisingly, the CD20-Ab itself might induce low levels of IFNs (lane 3), the result of which can be obtained consistently in two cell lines (data not shown). The data suggested that different virus can have the similar effects on IFN production upon CD20 treatments.

## Discussion

Innate immunity is important to control viral infection, but over-activation of the innate immunity may lead to autoimmune diseases. IFNs are a key component of host innate immunity. CD20 antibodies represent a class of successful drugs that used for treatment of lymphomas. Interestingly, IFN has been employed in the treatment of lymphomas with various degrees of success. Some clinical data have reported additive or synergistic activity of IFN with rituximab in treatment of lymphomas [45–47]. While many studies are centered the CD20 antibodies on tumor control, whether the antibodies have any effects on host innate immunity is unknown.

In this report, we studied the effects of CD20 antibody on innate immunity, specifically type I IFN productions. We find that: 1) CD20 antibody could potentiate the production of type I IFNs, and the potentiation is not related to viral replications (Figure 1); 2) The potentiation effect seems to be specific to B lymphocytes that express CD20 molecules (Figure 2); 3) The effects of CD20 antibody is apparently instant, and no pretreatments are needed (Figure 3); 4) The virus choices are not a factor for the potentiation effect (Figure 4B). All those data collectively indicates that CD20-Ab potentiates the production of type I IFNs in B lymphocytes.

Interestingly, CD20 antibody alone may induce low-levels of IFN-α (Figure 4B). The apparent differences between Figure 4B and Figures 1-3 in terms of IFN production may be due to the fact that only six hours treatment were used for Figures 1-3, but 24 hours were used for Figure 4B. In addition, a mixture of IFN-α subtypes, rather than IFN-β, was determined in Figure 4B. Of note, the clinical data suggest that the IFN pathways are activated in the CD20 antibody treatment [48–50]. Those data suggest that the CD20Ab may activate low levels of IFNs both in vivo and in vitro.

As TLR pathways are critical for lupus pathogenesis, we had tested if TLR3, TLR7, or TLR9 agonists (dsRNA, imiquimod, and ODN2395 respectively) and CD20 antibody for IFN induction, the enhancement by CD20-Ab was not observed (data not shown). It is known VSV-mediated IFN production is via TLR7 [44], but why TLR7 agonist (imiquimod) failed to induce IFNs in these B cell lines is not clear [51].

As a cellular gene, CD20 may have its own function. It is obvious that the function of CD20 is still elusive as one can delete the gene from mouse genome without obvious effect [52], and there is no ligand identified so far for the CD20 antigen. We suspect that the putative ligand binding to CD20, or the expression of CD20 alone, may be imitated and/or enhanced by CD20 and its Ab interactions. It is known that CD20 has calcium-channel activity and the function is stimulated by the CD20-Ab treatment [53]. In addition, ectopic expression of CD20 in a non-B cell line enhances IFN-β-promoter activity upon virus infection (Figure 4A). The data suggest that CD20 might be a component for IFN production in B lymphocytes.

The Rituximab was failed for the treatment of lupus patients with several explanations [20–25]. With our data in this report, we suspect that CD20 Ab may potentiate IFNs production in B lymphocytes in vivo by virus infections in lupus patients. Although a potentiation effect was not observed by TLR agonists (data not shown), lupus patients do have virus infections. For example, EBV is strongly associated with lupus and viral load is increased with the disease flares [54,55]. As IFNs are a key pathogenic factor for lupus pathogenesis, this research may provide possible mechanism for the failure of Rituximab in the treatment of lupus: the potentiation for IFN production as well as the low level induction of IFNs by CD20-Ab alone (Figures 1 and 4B). Although CD20-Ab may induce apoptosis in B lymphocytes, but at the same time, the IFN productions it might enhanced in vivo, may counteract the depletion effects of B cells. In essence, the report here may support the notion that anti-inflammatory strategies, not just B cell depletion, may be required for optimal therapy for SLE [25].

Rheumatoid arthritis (RA) is the most common chronic inflammatory disorder of the musculoskeletal system that may cause permanent joint damage. A beneficial role for type I IFN in RA has been identified [56,57]. Rituximab is approved worldwide for the treatment of RA, and highly beneficial in decreasing clinical symptoms, safe, and well tolerated. However, approximately 30-40% of RA patients do not respond to it. Genome-wide gene expression profiling of whole peripheral blood cells of RA patients shows that type I IFN response genes expression is associated with a good clinical response, whereas the IFN-response activity did not change or slightly decreased in the non-responders [48–50]. Our data suggest that an additional factor in RA patients may usurp the potentiation function and for the expression of IFN genes and therefore the IFN responsive genes.

In summary, we have discovered another function for the CD20 antibody, i.e., to potentiate B lymphocytes for type I IFN production. In the presence of virus infection, this potentiation function as well as the low level induction of IFNs by CD20-Ab alone (Figure 4B) suggest that a novel mechanism for the failure of the CD20-Ab treatment of lupus patients.
